# Seizure-Induced Periprosthetic Femoral Fracture After Total Hip Arthroplasty in a Patient With Epilepsy: A Case Report

**DOI:** 10.7759/cureus.84338

**Published:** 2025-05-18

**Authors:** Joshua L Dale, Zain Sayeed

**Affiliations:** 1 Osteopathic Medicine, William Carey University College of Osteopathic Medicine, Hattiesburg, USA; 2 Orthopedics, Doctors Hospital at Renaissance, Edinburg, USA

**Keywords:** adherence to medication, periprosthetic fractures, revision hip and knee surgery, seizure medications, total hip arthroplasty (tha)

## Abstract

Fractures secondary to seizure activity are a well-known complication in patients with epilepsy; however, periprosthetic fractures following total hip arthroplasty (THA) due to seizures are rarely documented. Nonadherence to anti-epileptic medications (AEMs) significantly increases the risk of seizure recurrence, particularly in individuals with a history of seizure-induced skeletal trauma. We present the case of a 27-year-old male with a documented history of epilepsy who sustained a right periprosthetic femoral fracture following a generalized tonic-clonic seizure. One year prior, the patient had undergone THA due to post-traumatic acetabular arthritis, which developed after a seizure-induced acetabular fracture. He later became noncompliant with his prescribed levetiracetam regimen, discontinuing use approximately one month after his initial THA. Imaging revealed a Vancouver B2 periprosthetic fracture with femoral stem subsidence. The patient underwent revision THA with open reduction and internal fixation and had an uneventful postoperative course. This case highlights the critical importance of medication adherence in patients with epilepsy, especially those with prior seizure-related orthopedic injuries. It also raises the need for further research into the long-term effects of levetiracetam on bone health. Regardless of the specific AEM, physicians should maintain a high index of suspicion for bone health deterioration and consider routine bone mineral density screening in this unique patient population.

## Introduction

Epilepsy is a relatively common neurological condition, affecting approximately 1.2% of the U.S. population [1]. Patients with epilepsy are at an increased risk for skeletal injuries due to both the biomechanical forces of tonic-clonic seizures and the risk of falling during episodes. Common seizure-related fractures include bilateral posterior shoulder dislocations, thoracic and lumbar vertebral compression fractures, and bilateral femoral neck fractures [2]. Though rare, acetabular fractures have also been reported following seizures, typically due to intense muscular contractions without direct trauma [3].

These injuries are clinically significant yet often underdiagnosed due to postictal confusion and the frequent absence of external trauma, which may obscure the clinical picture. Further complicating management, anti-epileptic medications (AEMs) have been shown to adversely affect bone metabolism. Older AEMs, such as phenobarbital, phenytoin, carbamazepine, and primidone, are known inducers of the cytochrome P450 system, accelerating vitamin D catabolism and thereby reducing bone mineral density (BMD) [4]. The impact of newer AEMs, such as levetiracetam, remains inconclusive. However, emerging evidence suggests that levetiracetam may significantly reduce serum calcium levels, raising concerns about long-term bone health in patients on chronic therapy [5].

While seizure-related fractures are well-documented, atypical fractures, such as acetabular fractures and central hip dislocations, remain underreported and present diagnostic challenges. This report highlights the risks associated with discontinuing seizure medications in patients with a history of prior fractures.

## Case presentation

**Figure 1:**
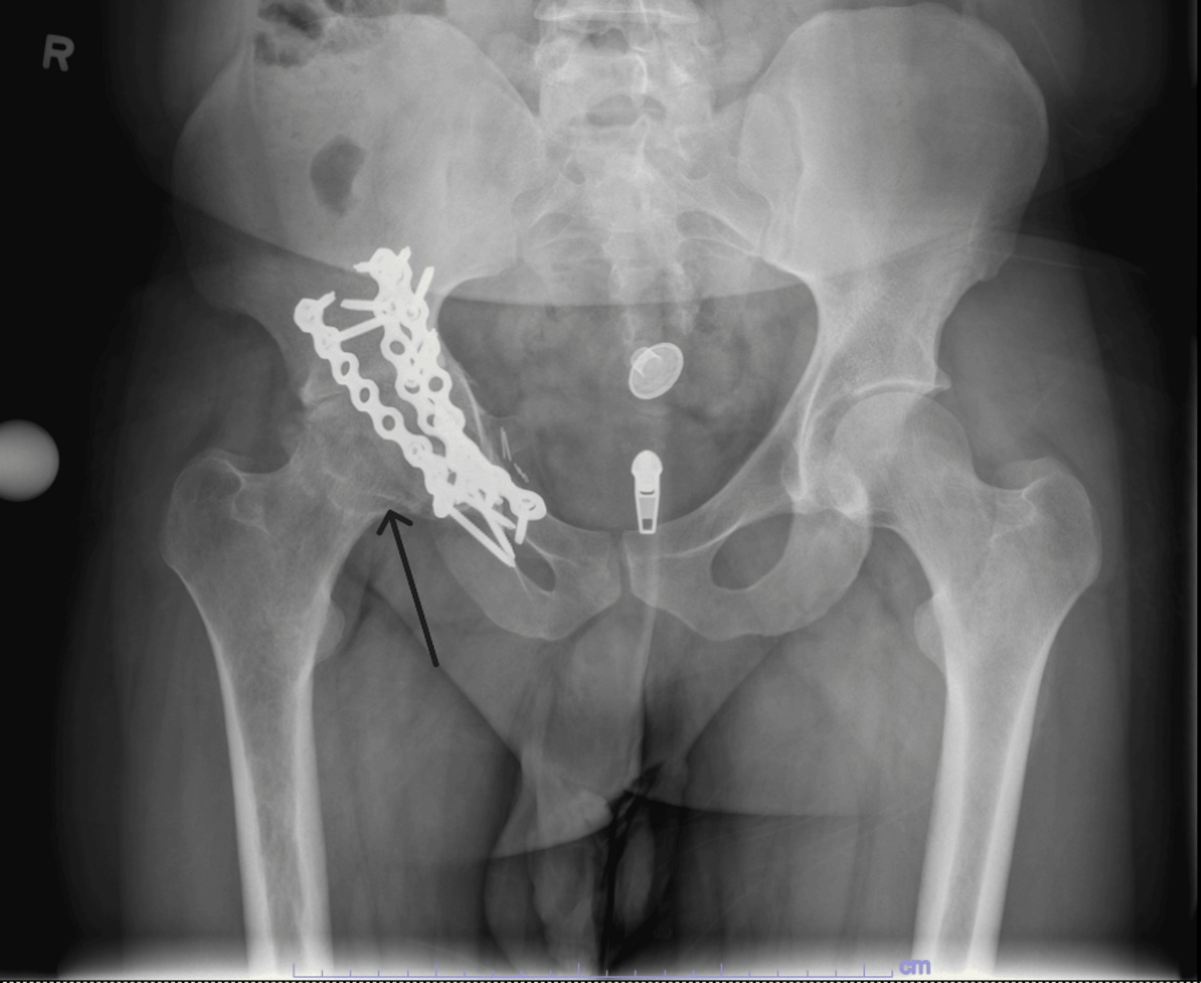
Preoperative AP X-ray of the pelvis demonstrating a femoral neck fracture with acetabular plating Arrow indicates the femoral neck fracture. AP, anteroposterior

**Figure 2:**
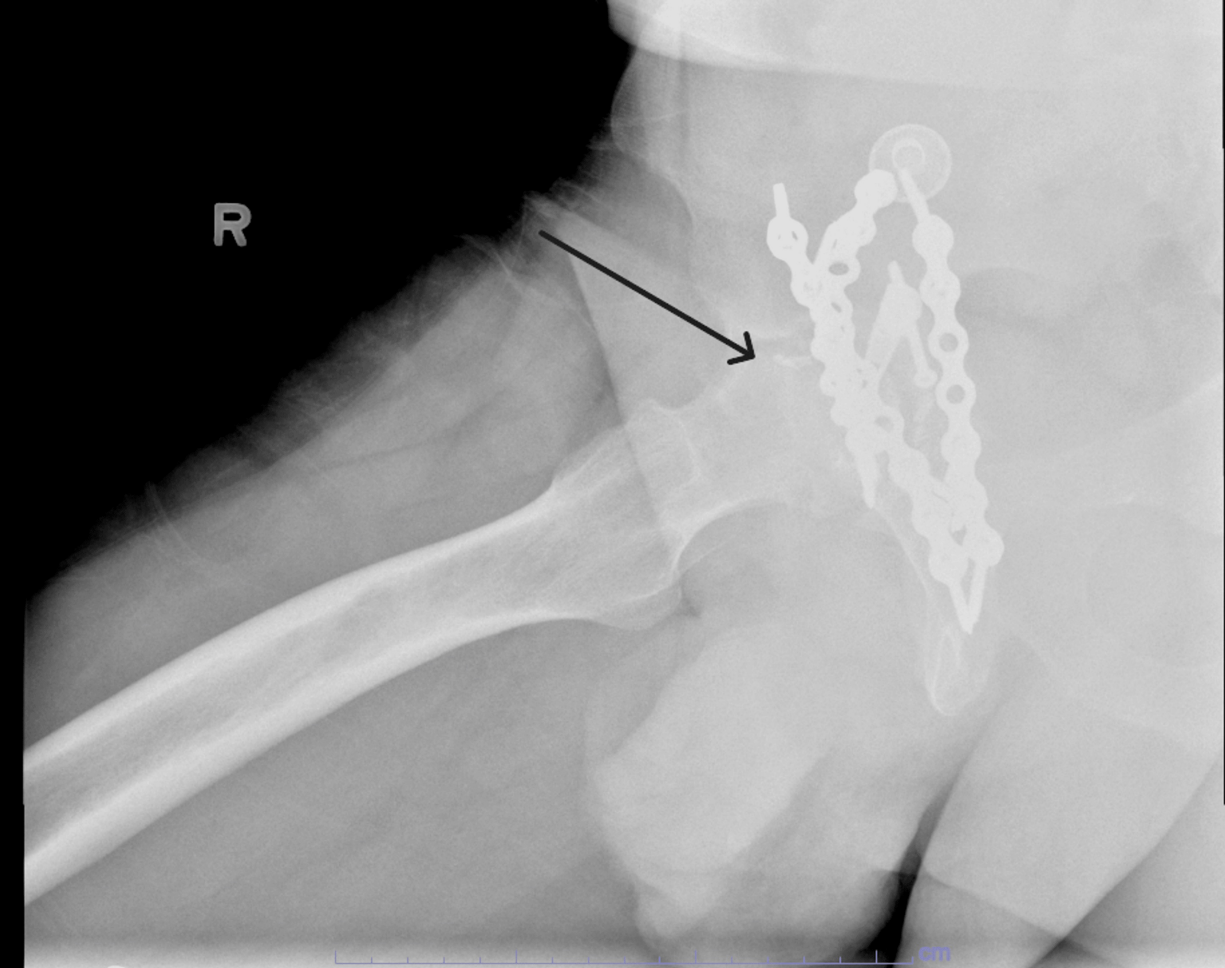
Cross-table lateral X-ray demonstrating a femoral neck fracture with acetabular plating Arrow indicates a femoral neck fracture

**Figure 3:**
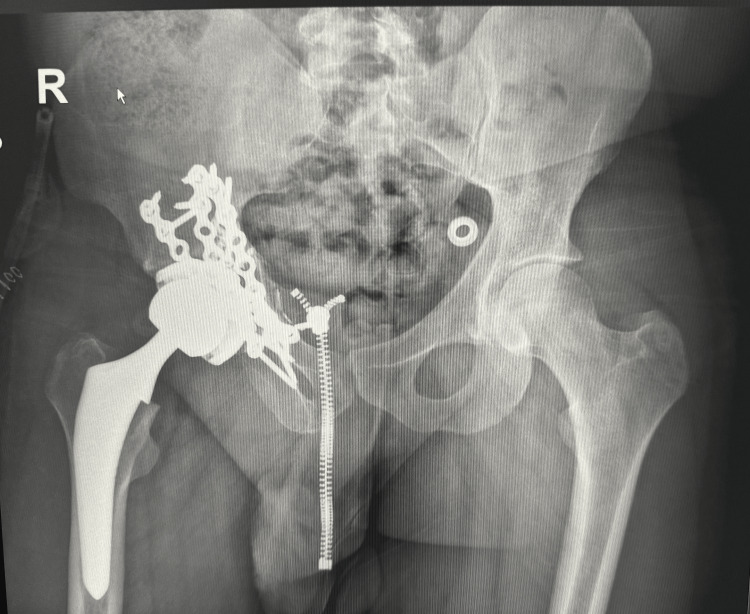
Postoperative AP pelvic X-ray of primary total hip arthroplasty AP, anteroposterior

A 27-year-old male with a known history of epilepsy was referred to our service from the medical floor due to severe right hip pain. One month prior, he had been evaluated for advanced acetabular arthritis secondary to a previous traumatic injury. Regarding social history, the patient reported smoking approximately five to nine cigarettes daily but denied alcohol consumption. His medical history included epilepsy with tonic-clonic seizures, managed with levetiracetam. The patient was first diagnosed with epilepsy five years ago, and his symptoms were initially controlled with levetiracetam. The patient experienced a breakthrough generalized tonic-clonic seizure during which he sustained a right acetabular fracture as his femoral head impacted the acetabulum. Two weeks prior to presentation, he had another breakthrough seizure, resulting in a right femoral neck fracture. The patient’s previous femoral neck fracture with plating is shown in Figures 1, 2. This recent injury, combined with advanced acetabular arthritis, led to the decision to perform a primary total hip arthroplasty (THA), as shown in Figure 3. The patient denied taking any medications other than levetiracetam.

During the current hospitalization, the patient reported nonadherence to his AEM, stating that he had discontinued levetiracetam for the prior two weeks. He attributed this lapse to a perceived improvement in his epilepsy, stating he had increased mental clarity, following his THA. Two days before admission, the patient reportedly had a seizure. The patient had no recollection of this event, but the patient's mother reported finding him on the floor when she returned home. The following day, the patient attempted to attend a physical therapy session for his initial THA but was unable to participate due to severe right hip pain, prompting him to present to the emergency department the next day.

On physical examination, the right lower extremity appeared shortened and externally rotated. Inspection of the left hip revealed no abnormalities. Sensation was intact bilaterally in both lower extremities. The patient also had a positive log roll test on the right side and a negative one on the left.

**Figure 4:**
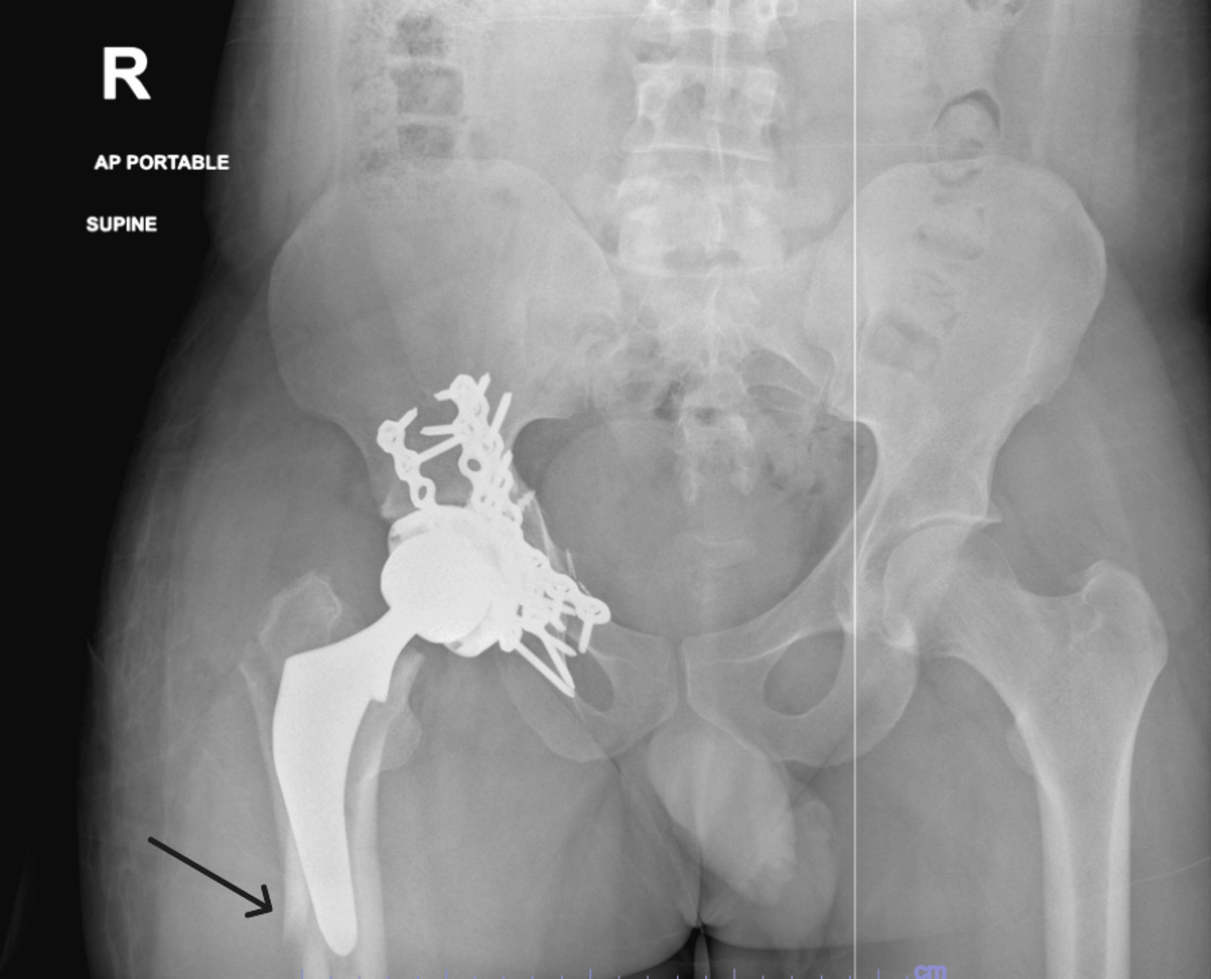
Preoperative AP X-ray of the pelvis demonstrating periprosthetic fracture of the femoral shaft Arrow indicated fracture around the prosthesis. AP, anteroposterior

**Figure 5:**
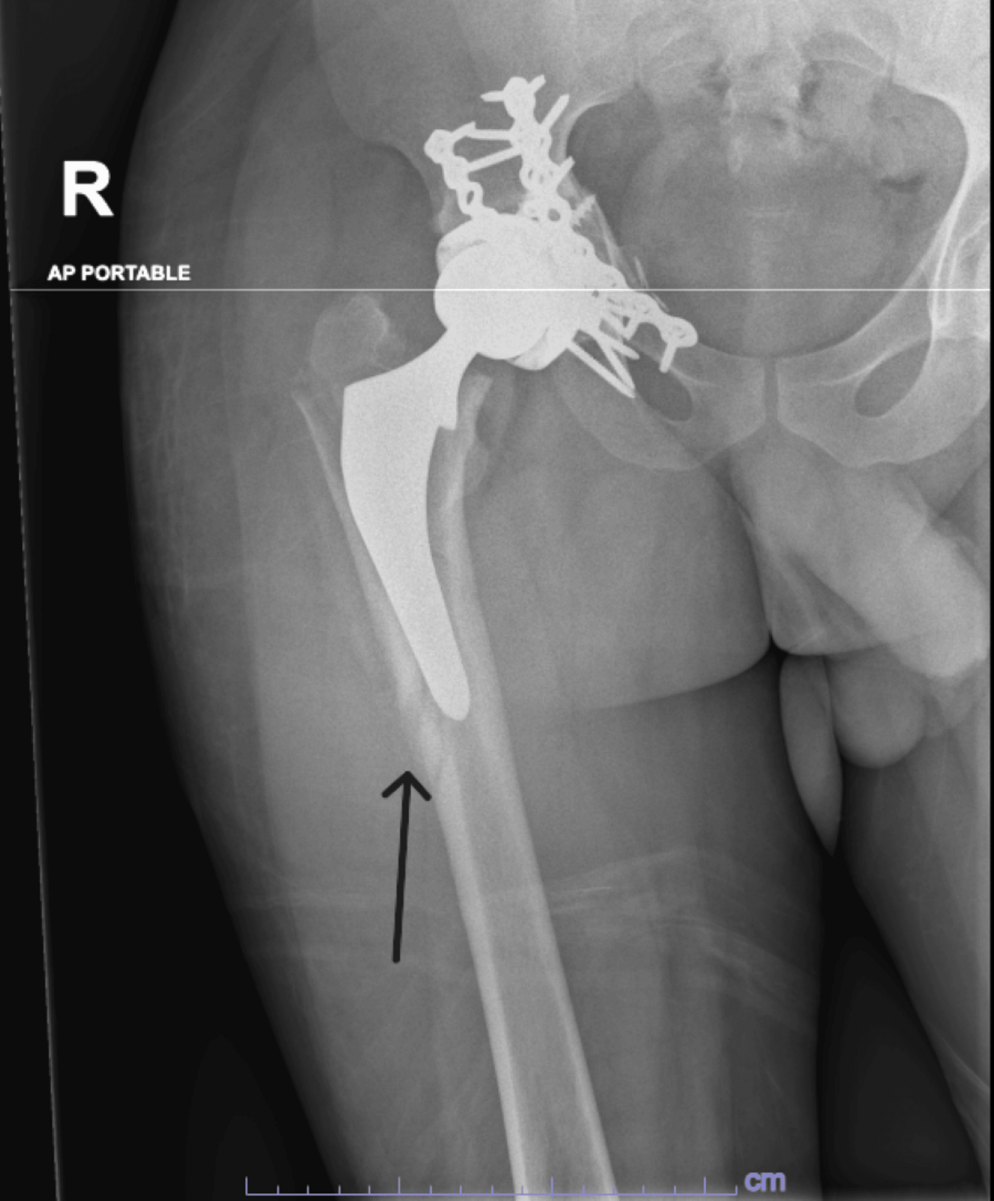
Preoperative AP X-ray of the right hip demonstrating periprosthetic fracture of the femoral shaft Arrow indicated fracture around the prosthesis. AP, anteroposterior

Imaging obtained in the emergency department revealed a Vancouver B2 periprosthetic femoral fracture, along with evidence of femoral stem subsidence. The patient’s periprosthetic fracture is shown in Figures 4, 5. The Vancouver classification has been discussed by Schopper et al., with a summary provided in Table 1 [6]. Based on the patient’s clinical presentation and radiographic findings, surgical intervention was recommended. We discussed in detail the risks and potential complications associated with revision THA and open reduction and internal fixation (ORIF) of the proximal femur fracture. The patient provided informed consent to proceed with surgery.

**Table 1:** Vancouver classification of periprosthetic fractures Table adapted from Schopper et al. [6]

Type	Subtype	Description
A	AG	Fracture of the greater trochanter
	AL	Fracture of the lesser trochanter
B	B1	Fracture around the stem; stem is stable; good bone stock
	B2	Fracture around the stem; stem is loose; good bone stock
	B3	Fracture around the stem; stem is loose; poor bone stock
C	-	Fracture well below the femoral prosthesis

With informed consent obtained, we proceeded with a right THA revision with ORIF of the femoral shaft. Intraoperatively, the periprosthetic femoral fracture was identified, reduced, and stabilized using cerclage cables due to lack of plates and screws for fixation.

**Figure 6:**
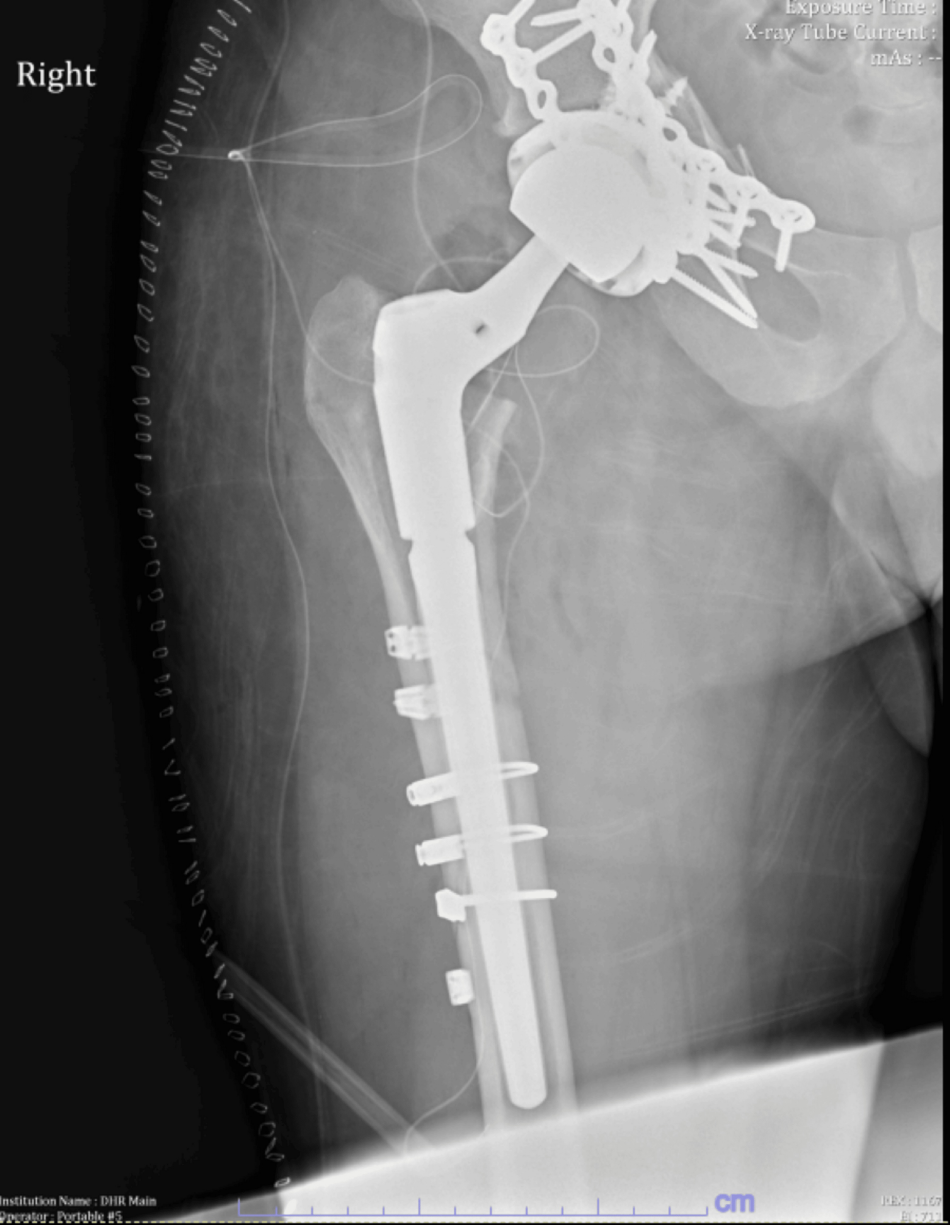
Postoperative AP X-ray of the right hip demonstrating long stem femoral prosthesis with a cerclage wire AP, anteroposterior

**Figure 7:**
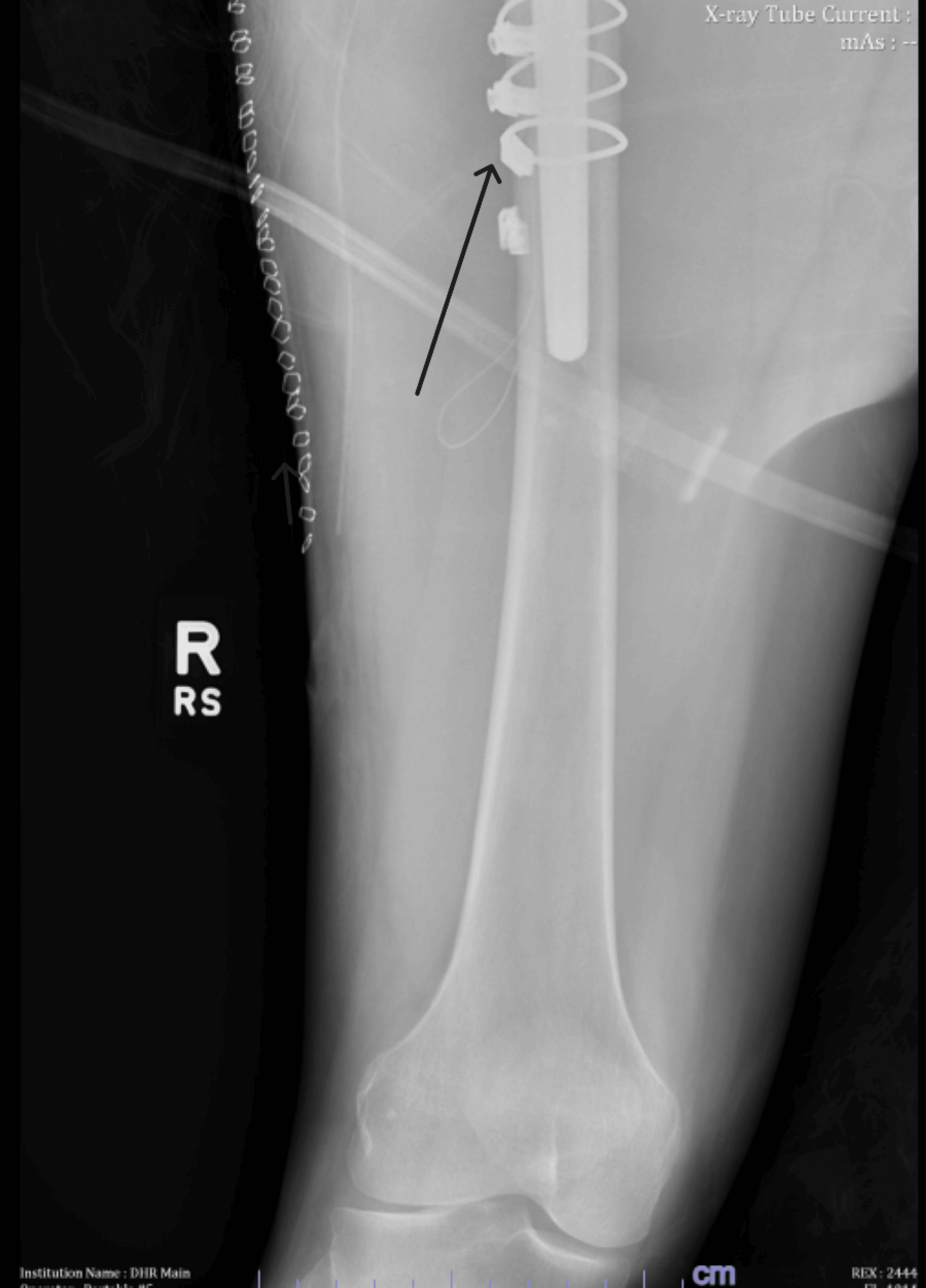
Postoperative AP X-ray of the right leg demonstrating the distal portion of the long stem femoral prosthesis Arrow indicates the cerclage wire AP, anteroposterior

After confirming appropriate alignment and positioning of the new prosthesis, standard layered closure was performed. The patient had an uneventful postoperative course and was discharged on postoperative day 3 with instructions to follow up in the outpatient clinic in 10 days. At the 10-day follow-up visit, the patient reported mild pain but noted that he was able to ambulate without significant difficulty. The postoperative film is displayed in Figures 6, 7.

Verbal consent was obtained from the patient to produce this case report due to the uniqueness of undergoing THA at a young age.

## Discussion

This case of THA is notable due to the patient's young age. The hip replacement was necessitated by trauma directly resulting from a seizure, a rare presentation in orthopedic practice. The uniqueness of this case is underscored by data indicating that more than 90% of primary THAs are performed in patients over the age of 50 [7].

A comparable case was reported by Grimaldi et al., involving a 49-year-old patient who sustained bilateral femoral neck fractures following a seizure [8]. While both patients suffered seizure-related hip trauma, key differences exist. In our case, the patient experienced a seizure after his initial arthroplasty, resulting in a periprosthetic femoral fracture that required revision surgery. In contrast, the patient in Grimaldi et al.’s report sustained their fractures as the primary injury. Most significantly, our patient was markedly younger, highlighting the exceptional nature of this presentation.

While older AEMs such as phenytoin and carbamazepine are well-documented in contributing to bone loss through cytochrome P450 enzyme induction, newer agents such as levetiracetam are not without risk. Emerging evidence suggests that levetiracetam may impair calcium metabolism, potentially leading to decreased BMD [5]. One study indicated that the short-term use of levetiracetam (less than one year) was associated with decreased bone density in the lumbar spine [9]. However, this study did not demonstrate significant changes in bone density with long-term use [9]. In animal studies, however, there is evidence of significant BMD loss. Research by Fekete et al. reported significant bone density reduction in both femurs of rats treated with levetiracetam [10]. Given the evidence of short-term bone loss in humans and significant bone loss in animal models, further studies are warranted to investigate the long-term effects of levetiracetam on BMD in humans.

Our patient, who had been taking levetiracetam for five years, believed that newer-generation antiepileptic drugs did not pose a significant fracture risk. This misconception underscores the need for improved education and routine monitoring of bone health in patients receiving long-term antiepileptic therapy.

Importance of medication adherence

This case also emphasizes the critical importance of medication adherence in patients with seizure disorders. According to Laue-Gizzi, abrupt discontinuation of AEMs is associated with a 46% risk of seizure recurrence, particularly within the first 6 to 12 months of cessation [11]. Our patient discontinued levetiracetam approximately two weeks after undergoing primary revision arthroplasty, which likely contributed to the rebound seizure and subsequent fracture requiring a second surgical intervention.

A study by Shiek Ahmad et al. further supports this concern, showing that the risk of fractures increases by approximately 4-6% with each additional year of AEM use [12]. Consequently, regular monitoring of calcium and vitamin D levels should be considered for all patients receiving chronic ASM therapy, regardless of the generation of the medication.

Surgical and postoperative management

The patient’s revision THA with ORIF of the proximal femur was an appropriate intervention given the presence of a periprosthetic fracture and evidence of femoral stem subsidence. Prior to surgery, the patient received IV cefazolin one hour before the procedure. The operation was performed using a posterior approach under spinal anesthesia. A modular stem was utilized, bypassing the fracture by two cortical diameters and cerclage fixation to enhance stabilization. The stem achieved a scratch fit of 4cm, a generally accepted standard for adequate femoral stability. Postoperatively, the patient was instructed to follow toe-touch weight-bearing precautions and standard posterior hip precautions, including the use of an abduction pillow. Pain management consisted of oral acetaminophen with intravenous hydromorphone for breakthrough pain. Postoperative antibiotic prophylaxis included cefazolin administered every eight hours for 48 hours due to excess drainage. On postoperative day 3, the patient was discharged in stable condition and prescribed a seven-day course of oral cefadroxil. A Prevena wound vacuum and dressing were applied, scheduled for removal at the patient’s 10-day follow-up.

## Conclusions

This case underscores the critical importance of medication adherence in patients with epilepsy. Our patient’s nonadherence to his prescribed anti-epileptic regimen following his initial THA significantly increased his risk for seizure recurrence and subsequent injury. Additionally, this case highlights the need for further research into the effects of levetiracetam on bone health. Currently, there is limited evidence evaluating its long-term impact on BMD, underscoring the importance of routine bone health monitoring in all patients receiving AEMs, regardless of the generation of the drug. Finally, this case emphasizes the need for improved patient education on the consequences of medication nonadherence, particularly in individuals with a history of seizure-related trauma.
